# Non-Invasive, Simultaneous Quantification of Vascular Oxygenation and Glucose Uptake in Tissue

**DOI:** 10.1371/journal.pone.0117132

**Published:** 2015-01-30

**Authors:** Narasimhan Rajaram, Andrew F. Reesor, Christine S. Mulvey, Amy E. Frees, Nirmala Ramanujam

**Affiliations:** Department of Biomedical Engineering, Duke University, Durham, North Carolina, United States of America; Mayo Clinic, UNITED STATES

## Abstract

We report the development of non-invasive, fiber-based diffuse optical spectroscopy for simultaneously quantifying vascular oxygenation (SO_2_) and glucose uptake in solid tumors *in vivo*. Glucose uptake was measured using a fluorescent glucose analog, 2-[N-(7-nitrobenz-2-oxa-1,3-diaxol-4-yl)amino]-2-deoxyglucose (2-NBDG). Quantification of label-free SO_2_ and 2-NBDG-fluorescence-based glucose uptake 60 minutes after administration of the tracer (2-NBDG_60_) was performed using computational models of light-tissue interaction. This study was carried out on normal tissue and 4T1 and 4T07 murine mammary tumor xenografts *in vivo*. Injection of 2-NBDG did not cause a significant change in optical measurements of SO_2_, demonstrating its suitability as a functional reporter of tumor glucose uptake. Correction of measured 2-NBDG-fluorescence for the effects of absorption and scattering significantly improved contrast between tumor and normal tissue. The 4T1 and 4T07 tumors showed significantly decreased SO_2_, and 4T1 tumors demonstrated increased 2-NBDG_60_ compared with normal tissue (60 minutes after the administration of 2-NBDG when perfusion-mediated effects have cleared). 2-NBDG-fluorescence was found to be highly sensitive to food deprivation-induced reduction in blood glucose levels, demonstrating that this endpoint is indeed sensitive to glycolytic demand. 2-NBDG_60_ was also found to be linearly related to dose, underscoring the importance of calibrating for dose when comparing across animals or experiments. 4T1 tumors demonstrated an inverse relationship between 2-NBDG_60_ and SO_2_ that was consistent with the Pasteur effect, particularly when exposed to hypoxic gas breathing. Our results illustrate the potential of optical spectroscopy to provide valuable information about the metabolic status of tumors, with important implications for cancer prognosis.

## Introduction

Hypoxia and deregulated cell metabolism are two hallmarks of cancer that play a significant role in tumor progression and invasion through proliferation, evasion of growth suppression, and resistance to cell apoptosis [1,2]. Although hypoxia is expected to lead to increased glycolytic demand in normal and cancer cells, certain cancer cells resort to aerobic glycolysis or metabolism of glucose to lactate even in the presence of oxygen. The transcription factor, hypoxia-inducible factor (HIF-1) and its downstream targets play an important role in this switch to aerobic glycolysis [3–6]. The switch to aerobic glycolysis has been shown to confer tumors with a growth advantage [7], and has been implicated in resistance to radiation and chemotherapy [8,9]. These recent findings have reiterated that measuring either oxygenation or glycolytic demand alone cannot necessarily provide a surrogate measure of the other. Thus, the relationship between oxygenation and glycolytic demand is important in informing the relationship between energy supply and demand and its association with tumor propensity for resistance or metastasis [10–12].

The classical approach to quantifying the relationship between oxygenation and metabolic demand *in vitro* involves measuring glucose uptake and lactate production in the presence and absence of oxygen. Pre-clinical animal studies typically involve immunohistochemistry of excised tumors stained with markers of hypoxia and glucose or GLUT1 [13]. Simultaneous measurements of both metabolism and oxygenation *in vivo* are possible through a combination of techniques—Electron paramagnetic resonance (EPR) imaging to quantify pO_2_ and hyperpolarized MRI using ^13^C-labeled glucose to monitor its intracellular fate [14,15]. Positron Emission Tomography (PET) of FDG uptake is used extensively in the clinic to exploit increased glucose uptake of the tumor and hence identify tumor location within the body. However, PET imaging of hypoxia markers, such as FMISO has suffered from poor specificity and signal to noise ratio [16]. Given the dynamic nature of changes in a tumor microenvironment and its role in determining long-term tumor clinical outcome [17–19], it is important to develop functional imaging approaches that can repeatedly measure both oxygenation and glucose uptake *in vivo*. Such repeated measurements could provide insight into the transient changes in tumor biology and identify windows of opportunity for therapeutic interventions.

Optical measures of vascular oxygenation (SO_2_) in a window chamber have been shown to be concordant with hypoxia measurements performed using oxygen-sensing nanoparticles [20,21]. 2-[N-(7-nitrobenz-2-oxa-1,3-diaxol-4-yl)amino]-2-deoxyglucose (2-NBDG) is a fluorescent glucose analog whose uptake in breast cancer cells is GLUT-1-dependent and sensitive to glycolytic perturbations, such as treatment with tamoxifen [22]. We have demonstrated with intra-vital microscopy, imaging of SO_2_ and uptake of 2-NBDG in mammary tumors growing in a dorsal skin-flap window chamber [23]. Our results initially revealed that 4T07 tumors were better oxygenated than the 4T1 tumors and mean 2-NBDG-uptake was significantly higher in the 4T1 tumors. However, detailed analysis revealed that both 4T1 and 4T07 tumors demonstrated distinct patterns of 2-NBDG uptake that depended on the rates of delivery and clearance of 2-NBDG that were, in turn, dependent on tumor SO_2_. Specifically, the rate of delivery of 2-NBDG (R_D_) was lowest in severely hypoxic regions, leading to low 2-NBDG uptake. For increasing R_D_ values that were lower than the glucose consumption rate, 2-NBDG uptake increased with R_D_. However, when we increased R_D_ beyond the glucose consumption rate by inducing hyperemia, we observed a decrease in 2-NBDG uptake. We recently showed that the delivery rate of 2-NBDG was correlated with red blood cell velocity, thus establishing a link between 2-NBDG uptake and blood flow velocity [24]. These results established the importance of measuring both 2-NBDG kinetics and SO_2_ in order to accurately interpret glucose uptake data from tumors *in vivo*.

Although high-resolution intra-vital microscopy provides a powerful pre-clinical tool to quantify the relationship between SO_2_ and glucose uptake in the tumor microenvironment, it is not readily translatable to the characterization of solid tumors. To address this issue, we have developed a quantitative optical spectroscopy technique to measure the scattering, absorption and fluorescence of turbid media such as thick tissues [25,26]. Specifically, we have developed a series of fast, scalable, Monte Carlo (MC) inverse models that model light-tissue interaction for the specific optical fiber-probe geometry used for measurement with the optical spectroscopy device. The MC reflectance model uses the spectral features of known scattering and absorption components in tissue to adaptively fit the measured diffusely reflected light and extract quantitative parameters that report on tissue scattering, hemoglobin concentration ([THb]), and SO_2_ [25,27]. Additionally, the MC fluorescence model quantifies the intrinsic or native fluorescence of fluorophores in tissue [26,28] using the measured fluorescence and the extracted absorption and scattering properties because both the excitation light and the fluorescence emission are distorted by tissue scattering and absorption. We have previously applied this technology to characterize the SO_2_ and [THb] of pre-malignant and malignant tissue in both pre-clinical models [29,30] and human subjects [31–33]. Measurements of SO_2_ have been shown to be strongly associated with tissue pO_2_ [34]. We have also quantified 2-NBDG concentrations in tissue-simulating phantoms that contained either single or multiple fluorophores and demonstrated that the MC fluorescence model effectively removes the non-linear effects of absorption and scattering on the fluorescence, thus yielding a linear relationship between 2-NBDG fluorescence intensity and concentration [28].

The objective of the current study was two-fold: 1. Demonstrate the ability of a fast and non-invasive method to simultaneously measure SO_2_ and glucose uptake (using 2-NBDG) in normal tissues and in solid tumors in pre-clinical models *in vivo*, and 2. Characterize differences in the relationship between SO_2_ and glucose uptake in 2 sibling murine breast cancer lines that differ in their metastatic potential as well as normal, non-tumor bearing tissue. We assessed the sensitivity of 2-NBDG-fluorescence to food deprivation-induced changes (negative perturbation) and injected dose (positive perturbation). We characterized SO_2_ and 2-NBDG_60_ in all three tissue-groups (normal tissues, 4T1 and 4T07 tumors) and compared the results to *in* vitro metabolic assays. Finally, we exposed 4T1 tumors to hypoxia and examined the relationship between 2-NBDG and SO_2_ for a wide range of SO_2_ values and compared these findings to both our previous dorsal skin-flap window chamber studies. Our results illustrate the potential of optical spectroscopy as a valuable pre-clinical and clinical tool for simultaneously quantifying two important hallmarks of cancer, whose relationship has important implications for cancer progression, metastasis, and treatment resistance.

## Methods

### Ethics Statement

This study was carried out in accordance with the recommendations in the Guide for the Care and Use of Laboratory Animals of the National Institutes of Health. The protocol was approved by the Duke University Institutional Animal Care and Use Committee (Protocol Number: A255–12–09). All experiments were performed under isoflurane gas anesthesia, and all efforts were made to minimize suffering.

### Cell Culture Maintenance and Seahorse Assay

Two murine mammary carcinoma cell lines, 4T1 and 4T07, were used in this study. 4T1 cells were acquired from the ATCC and the 4T07 cells were generously provided by the laboratory of Dr. Mark Dewhirst at Duke University. Though arising from the same tumor, the cell lines have distinct metastatic properties [35,36]. 4T1 cells have been shown to metastasize throughout the body to organs such as the lung, liver, bone and brain. 4T07 is less invasive, spreading to the lung and liver but failing to form metastatic nodules. Both cell lines were cultured in Dulbecco’s Modified Eagle Medium (DMEM, Gibco, Carlsbad, California) supplemented with 10% fetal bovine serum and 1% antibiotics and kept free from contaminants. Cells were passaged every 2–3 days and kept incubated at 37.0°C and 5.0% O_2_.

A Seahorse Glycolytic Stress Test [Seahorse Biosciences, Massachusetts, USA] was used to probe the metabolic properties of 4T1 and 4T07 cells. Oxygen consumption rate (OCR) and extracellular acidification rate (ECAR) were measured every 11 minutes. Between minute 22 and minute 33 of the assay, 25 mM glucose was injected to each well. Between minute 55 and minute 66, 1 μM oligomycin was injected to each well. Results for each well were normalized to the number of cells in each well. Results represent the average of 12 total wells for each cell line: assays were performed on 3 different days and each assay contained 4 wells of each cell line.

### *In vivo* studies

Female athymic nude mice, 8 to 10 weeks old (nu/nu, NCI, Frederic, Maryland) and weighing between 20 and 25 g, were used for these studies. All animals were housed in an on-site facility with *ad libitum* access to food and water and standard 12-hour light/dark cycles. Normal mice with no tumors were used for the fasting and dosage experiments. Groups of five mice were fasted for 6 hours and injected with 6, 12, 24, or 36 mM of 2-NBDG (2–16 mg/ml or 8–48 mg/kg of body weight). For the fasting experiments, spectroscopic measurements of reflectance and fluorescence were performed in the morning on non-fasted mice. 2 days later, mice were fasted overnight for 12 hours prior to spectroscopic measurements. Blood glucose levels were measured by a tail-vein prick using a commercially available blood glucose meter (Freestyle), prior to optical measurements. Mice were not anesthetized during measurement of blood glucose to avoid effects of isofluorane on initial blood glucose levels. During the 12-hour fasting period, animals were only provided water.

A separate group of 10 mice received a subcutaneous injection of 4T1 (N = 5) or 4T07 (N = 5) cells in the right flank. The growth of these 4T1 and 4T07 tumor xenografts does not require estrogen injections. Once cells in culture reached 70% confluence, cells were trypsinized with trypsin-EDTA and suspended in serum-free medium. Cells were counted using a hemocytometer and drawn into an injection syringe at a concentration of 1 million cells in 0.1 ml. The mouse was anesthetized using a mixture of isofluorane and room air (1.5% v/v). The skin above the mouse’s leg was wiped with alcohol, and 0.1 ml of the cell solution was injected subcutaneously. Mild pressure was applied for a few seconds to the injection site to prevent leakage of the solution. Mice were removed from anesthesia, weighed, and returned to the cage. The entire procedure typically takes 1–1.5 minutes. Mice were monitored continuously over the next two weeks for signs of tumor growth. Tumor volume was measured using the short (a) and long (b) dimensions of the tumor as (π x a^2^ x b/6). Once the tumor volume was approximately 100 mm^3^, mice were fasted for 6 hours, anesthetized with isofluorane, and injected with 100 μl of 2-NBDG (6 mM or 8 mg/kg). Normal mice and 4T1-tumor-bearing mice in the normoxic group were exposed to isofluorane mixed with room air, and mice in the hypoxic group were exposed to isofluorane mixed with 10% Oxygen-90% Nitrogen. Non-tumor-bearing mice in the saline group (N = 3) were injected with 100 μl of phosphate-buffered saline (PBS).

### Optical measurements

The optical spectroscopy instrument has been described previously [37] and consists of a 450 Watt Xenon lamp coupled to a monochromator (Jobin Yvon Horiba), a fiber-optic probe (designed in-house and custom built by RoMack Inc.), a spectrograph (Jobin Yvon Horiba), and a 2D CCD camera (Jobin Yvon Horiba). The fiber-optic probe consisted of 19 illumination fibers (diameter = 200 μm; NA = 0.22) surrounded by 18 collection fibers (diameter = 200 μm; NA = 0.22). The sensing depth of the probe was estimated from tissue-mimicking phantoms to be approximately 1.5 mm.

The optical instrument was always allowed to warm up for at least 30 minutes before initiating measurements. The optical probe was stabilized to avoid probe bending—associated changes in lamp throughput and systematic errors [38]. Because changes in lamp throughput could affect optical measurements, reflectance and fluorescence spectra on each day were calibrated using a 20% reflectance standard (Spectralon, Labsphere) and a fluorescence standard (USF 210–010, Labsphere Inc.), respectively. Specifically, tissue reflectance spectra were divided, wavelength-by-wavelength, by the reflectance spectrum measured from the standard. The reflectance standard measurement also corrects the tissue reflectance spectra for the wavelength response of different system components. Fluorescence spectra were divided by the fluorescence intensity at 540 nm measured from the fluorescence standard. To correct the fluorescence spectra for wavelength response, the fluorescence spectrum from a NIST-approved tungsten calibration lamp (Optronic Laboratories Inc., Orlando, FL) was measured using the optical instrument and divided by the manufacturer-provided spectrum to obtain a correction factor. Tissue fluorescence spectra were multiplied by this correction factor to calibrate the wavelength-dependent response of the monochromators, fiber bundle and PMT. Because 1–3 mice were imaged on a given day for a total duration of 5 hours, standard measurements were performed prior to optical measurements on each mouse.

Mice were anesthetized using a mixture of isofluorane and room air (1.5% v/v) throughout the course of the optical measurements. Optical measurements on normal, non-tumor-bearing mice were obtained by placing the fiber-optic probe on the skin covering the right leg of the mouse on top of the thigh muscle. This position was selected because tumor-bearing mice had tumors implanted in this location. The same location was used for all mice. Prior to 2-NBDG injection, baseline reflectance and fluorescence spectra were measured from the tissue site of interest. All measurements for both phantom and animal studies were acquired in a dark room. Reflectance spectra were acquired from 390–650 nm (acquisition time: 0.05 s) and fluorescence emission spectra were acquired from 510–620 nm (acquisition time: 5 s) using excitation at 490 nm. Although 2-NBDG is maximally excited at ~ 475 nm, an excitation wavelength of 490 nm was used to minimize fluorescence excitation of endogenous FAD. Background subtraction was performed at the same integration time for each reflectance and fluorescence measurement, leading to a total integration time of 0.1 s and 10 s, respectively. Optical measurements on each mouse were acquired continuously for a period of 75 minutes. Specifically, 5 reflectance measurements and 175 fluorescence measurements were acquired over the 75-minute period. All data were acquired from a single spot—normal tissue or tumor. The probe was stabilized with a clamp and care was taken to ensure that pressure was not applied on tissue. Switching between white light illumination for reflectance and a single 490 nm illumination for fluorescence took nearly 50 s. Fluorescence measurements over the 75-minute period were collected as follows – 3 sets of 25 spectra followed by 2 sets of 50 spectra (3 x 25 + 2 x 50 = 175 spectra). Each set was preceded by a single reflectance measurement (1 x 5 = 5 spectra). No averaging was performed on the spectra.

### Measurement of tissue optical properties and native fluorescence

**Fig 1:**
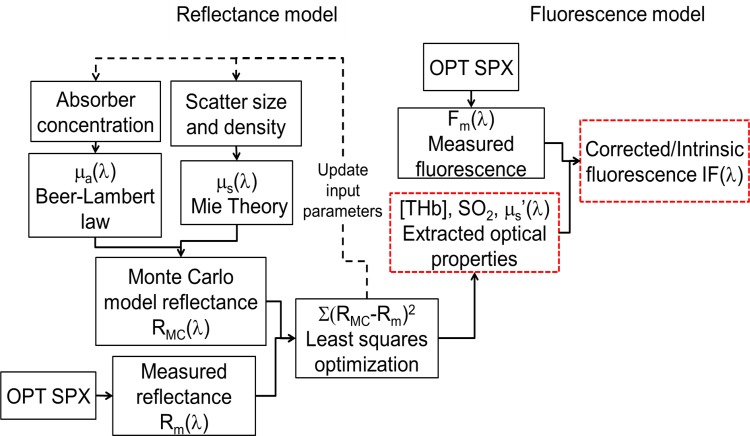
Flowchart illustrating the working of the MC reflectance and fluorescence models to extract optical properties and distortion-free fluorescence from tissue. μ_a_(λ) and μ_s_(λ) refers to the absorption and scattering coefficients, respectively.

A scalable inverse Monte Carlo model was used to extract tissue scattering, absorption and native fluorescence of 2-NBDG from *in vivo* optical measurements. The reflectance and fluorescence-based inverse Monte Carlo models have been described in detail previously [25–27]. Further, the fluorescence model has been validated for both single and multiple fluorophores in the sampled medium [28]. A flowchart describing the entire process is presented in **Fig. 1**. Because the Monte Carlo model operates on an absolute scale and the tissue measurements are relative to a reflectance standard, a reference phantom with known optical properties is necessary to accurately scale tissue optical properties. Based on a series of phantom studies using the optical instrument and fiber-optic probe described here, a reference phantom was selected based on low errors in extracting tissue absorption and scattering (described in next section). The inverse model assumes oxygenated hemoglobin, deoxygenated hemoglobin, and overlying rat skin as absorbers, and utilizes the widely used extinction coefficients documented by Scott Prahl to calculate absorption coefficients (units of cm^-1^). Tissue scattering is assumed to be primarily due to cells and cellular components and is calculated from scatterer size, density, and the refractive index of the scatterer and surrounding medium using Mie theory for spherical particles. The inverse model works by adaptively fitting the modeled diffuse reflectance to the measured tissue diffuse reflectance until the sum of squares error between the modeled and measured diffuse reflectance is minimized.

### Selection of reference tissue-mimicking phantom

Two sets of tissue-mimicking phantoms with varying scattering and absorption levels were prepared. Each phantom consisted of deionized water, hemoglobin (H0267, Sigma-Aldrich, St. Louis, MO) as the absorber and 1-μm monodisperse polystyrene spheres (07310, Polysciences, Warrington, PA) as the scatterer. The absorption spectrum of stock hemoglobin solution was measured using a spectrophotometer (Cary 300, Varian, Inc.), and used to determine the final absorption of the phantom. The scattering levels in the stock polystyrene sphere solution were calculated from Mie theory for spherical particles. A mixture of deionized water and polystyrene spheres was used to generate initial reduced scattering coefficients of 11 and 22 cm^-1^, respectively in two phantoms. Within each phantom, 6 increasing concentrations of hemoglobin were added to generate absorption coefficients of 1.25–8.1 cm^-1^. Hemoglobin concentration was increased by adding aliquots of the stock hemoglobin solution (approximately 5% of total phantom solution). The addition of this aliquot also resulted in decreasing scattering levels for each phantom (21.24–15.93 cm^-1^ and 10.82–8.12 cm^-1^) that were taken into account while calculating the errors in recovering scattering and absorption values. After each addition of hemoglobin, diffuse reflectance spectra were measured from the phantom. The diffuse reflectance from each phantom was used as a reference phantom in the MC inverse model to extract the scattering and absorption values of the other 11 phantoms. The tissue-mimicking phantom that generated the lowest error in extracting scattering and absorption was selected as the reference phantom for the *in vivo* study. The selection of reference tissue-mimicking phantoms has been described elaborately by Bender et al. [27].

### Quantification of glucose uptake

We have previously shown using confocal microscopy of dorsal skin flap window chambers that 2-NBDG uptake *in vivo* in tumors overlaps with tumor-positive areas 60 minutes after 2-NBDG injection [23]. Therefore, we used the fluorescence intensity at 60 minutes – 2-NBDG_60_ to indicate glycolytic demand by the tumor.

### Statistics

Comparison of mean values between groups for statistical significance was performed using Wilcoxon rank sum or Wilcoxon sign rank tests depending on the dataset. Pearson’s correlation coefficient was calculated to assess relationship between variables. MATLAB was used to perform all statistical calculations.

## Results

### Optical measures of SO_2_ are significantly lower in tumors compared with normal tissue

**Fig 2:**
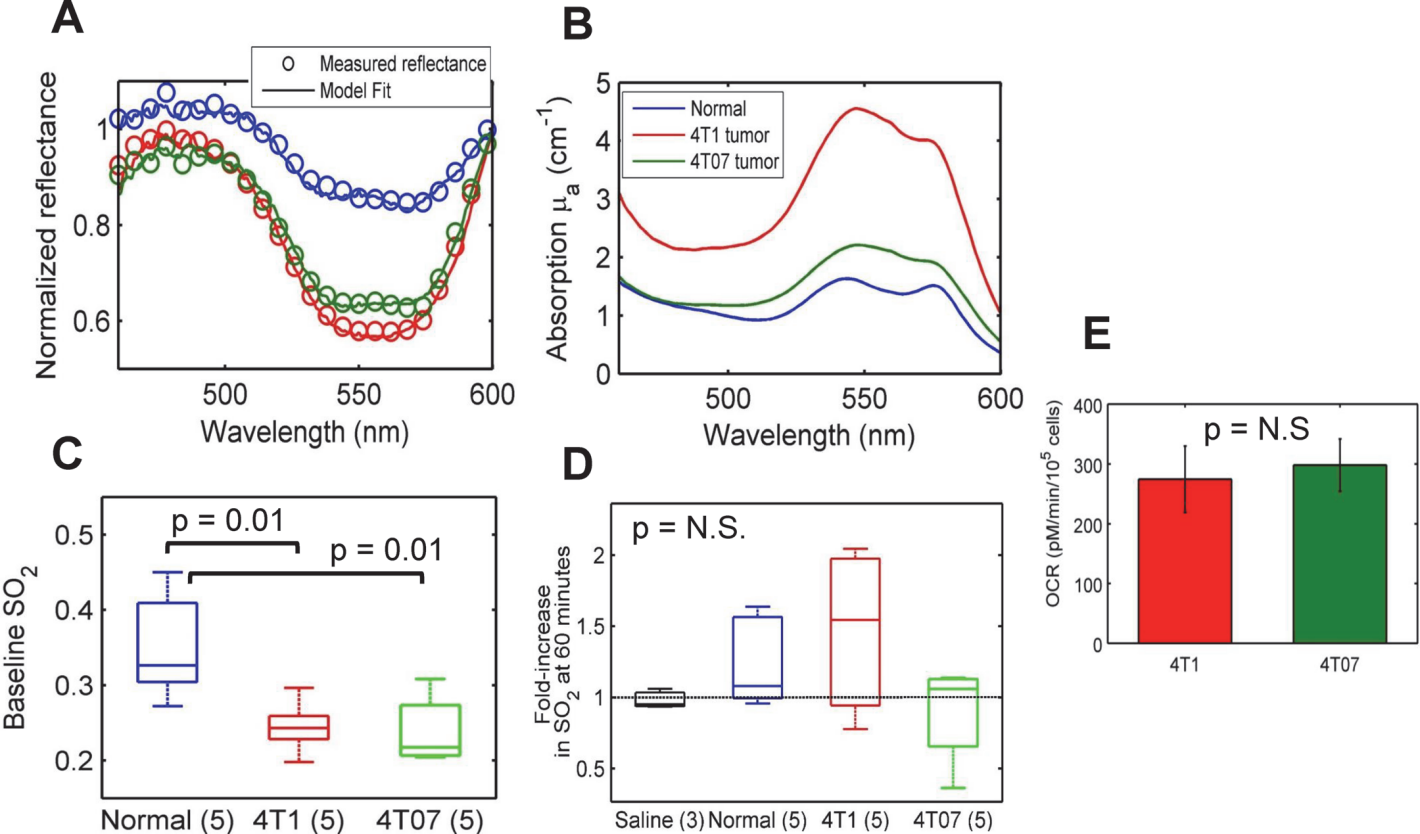
Optical measures of SO_2_ are significantly lower in tumors compared with normal tissue. **A**. Representative reflectance spectra (open circles) from normal tissue (blue), 4T1 (red) and 4T07 (green) murine mammary tumors, and MC model fits (solid line). These spectra were measured prior to 2-NBDG injection **B**. Extracted absorption spectra for the reflectance spectra shown in 2A illustrate higher absorption in the 4T1 tumor compared with the normal and 4T07 tumor. **C**. Baseline oxygenation levels are significantly lower in 4T1 and 4T07 tumors compared with normal tissue. **D**. The injection of 2-NBDG causes a statistically insignificant increase in SO_2_ in normal tissue and 4T1 tumors, and no change in the control and 4T07 groups **E**. Oxygen consumption rates (OCR) of 4T1 and 4T07 cells are statistically similar. Measurements were made using a Seahorse Glycolysis stress test. Data represent n = 12 cell samples from 3 distinct assays. Error bars represent standard error of the mean.

**Fig. 2A** shows representative reflectance spectra (normalized to 600 nm) measured from three different groups of mice—non-tumor bearing and 4T1 and 4T07 murine mammary tumor xenografts. For all groups, the representative spectra were measured prior to tail-vein injection of a 6 mM (2 mg/ml) dose of 2-NBDG. Open circles represent the measured reflectance and the solid lines represent the MC reflectance model fits to the data. Based on these model fits, the extracted absorption spectrum for each reflectance measurement is presented in **Fig. 2B**. The absorption levels are significantly higher in the 4T1 tumor compared to that in normal tissue and the 4T07 tumors. SO_2_ levels, calculated from the relative concentrations of oxygenated and deoxygenated hemoglobin, are significantly lower in the 4T1 and 4T07 tumors compared with normal tissue (**Fig. 2C**). The low levels of SO_2_ (30–45%) in normal tissue are likely due to the sampling location of the optical probe—skeletal muscle tissue, where oxygenation levels are known to be lower [39,40]. **Fig. 2D** shows the effect of 2-NBDG injection on SO_2_ within tissue and tumors. A small but insignificant increase in SO_2_ was observed in normal tissue and the 4T1 tumors at 60 minutes relative to baseline (prior to injection), which is the time point at which 2-NBDG-fluorescence stabilizes from transient perfusion-mediated effects. A small but insignificant change in SO_2_ was observed in the 4T07 tumors. Injection of saline did not cause a significant change in SO_2_. Furthermore, the average fold-increase in SO_2_ was not statistically different between any of the groups. The fold-increase in SO_2_ was not significantly correlated with 2-NBDG-fluorescence at 60 minutes within normal tissue or tumors (**data not shown;** r = 0.07; p = 0.78). The oxygen consumption rates of both cell lines were calculated using a glycolysis stress test, and were found to be statistically similar (**Fig. 2E**).

### Intrinsic tissue fluorescence corrected for absorption and scattering shows a significant increase in 2-NBDG_60_ in 4T1 tumors relative to normal tissue

**Fig 3:**
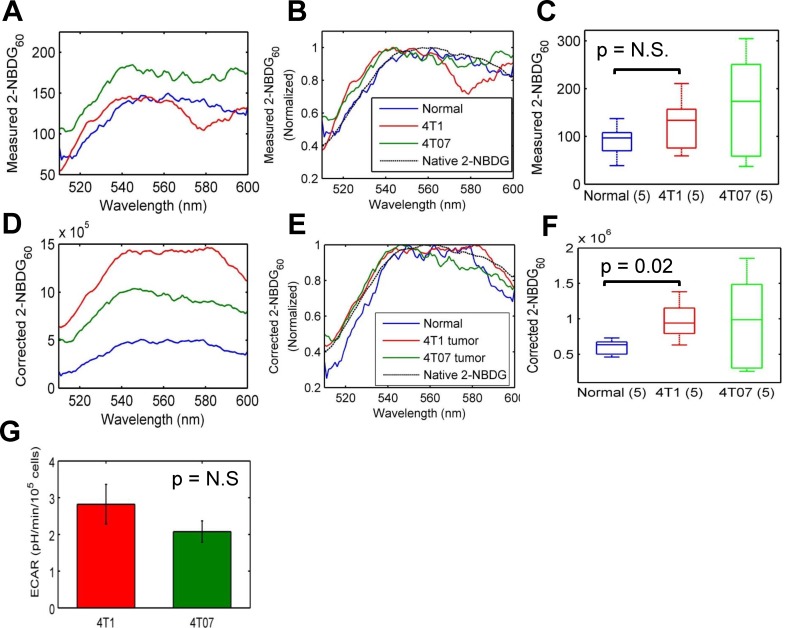
Intrinsic tissue fluorescence corrected for absorption and scattering improves 2-NBDG contrast between 4T1 tumors and normal tissue. **A**. Measured 2-NBDG_60_ from the 4T1 and 4T07 tumors is distorted by hemoglobin absorption, and is on par with fluorescence from normal tissue. A normal tissue data-point and representative tumors with similar measured fluorescence values were selected to illustrate the effect of correction. **B**. Normalized spectra of measured 2-NBDG_60_ illustrate the distortion in better detail. **C**. Measured 2-NBDG_60_ is not significantly different between the different groups. **D**. Correction with the MC fluorescence model removes hemoglobin-induced distortions and improves contrast between normal and tumor. **E**. Corrected 2-NBDG_60_ spectra from normal tissue, a 4T1 tumor, and a 4T07 tumor shown in 2D, normalized to their respective maxima are presented along with a true 2-NBDG fluorescence measurement, illustrating good agreement between the extracted in vivo spectral line shapes and native 2-NBDG. **F**. Corrected 2-NBDG_60_ is significantly higher in 4T1 tumors compared with normal tissue (p = 0.02). Although mean 2-NBDG_60_ in 4T07 tumors is higher compared with normal tissue, this is not statistically significant. **G**. The extracellular acidification rate (ECAR) of 4T1 and 4T07 cells, as calculated with a Seahorse Glycolysis stress test, is not significantly different. Data represent n = 12 cell samples from 3 distinct assays. Error bars represent standard error of the mean.

Measured 2-NBDG-fluorescence spectra at 60 minutes are shown in **Fig. 3A**. The fluorescence intensities from three representative mice (normal, 4T1, and 4T07) are similar; however, there is a visible distortion of the fluorescence line shape measured from the 4T1 tumor in the wavelength range corresponding to hemoglobin absorption (560–600 nm). Normalized versions of the same spectra illustrate the effect of distortion in better detail (**Fig. 3B**). There are no significant differences in 2-NBDG_60_ between the three groups (**Fig. 3C**). Correction with the MC model removes the distortion due to hemoglobin from the fluorescence line shape, and the recovered line shape is a closer representation of the actual 2-NBDG spectrum (**Fig. 3E**). Corrected 2-NBDG_60_ is significantly higher in the 4T1 tumors compared with normal tissue (**Fig. 3F**). Although the median value of the 4T07 tumors was similar to that of the 4T1 tumors, there was no significant difference in 2-NBDG_60_ between the 4T07 tumors and the other two groups. This was primarily due to the large variance in fluorescence intensity in the 4T07 group in both the measured and corrected forms. Analysis of the extracellular acidification rate (ECAR) using glycolysis stress tests showed no significant differences between 4T1 and 4T07 cells (**Fig. 3G**).

### 2-NBDG_60_ increases with a decrease in blood glucose levels

**Fig 4:**
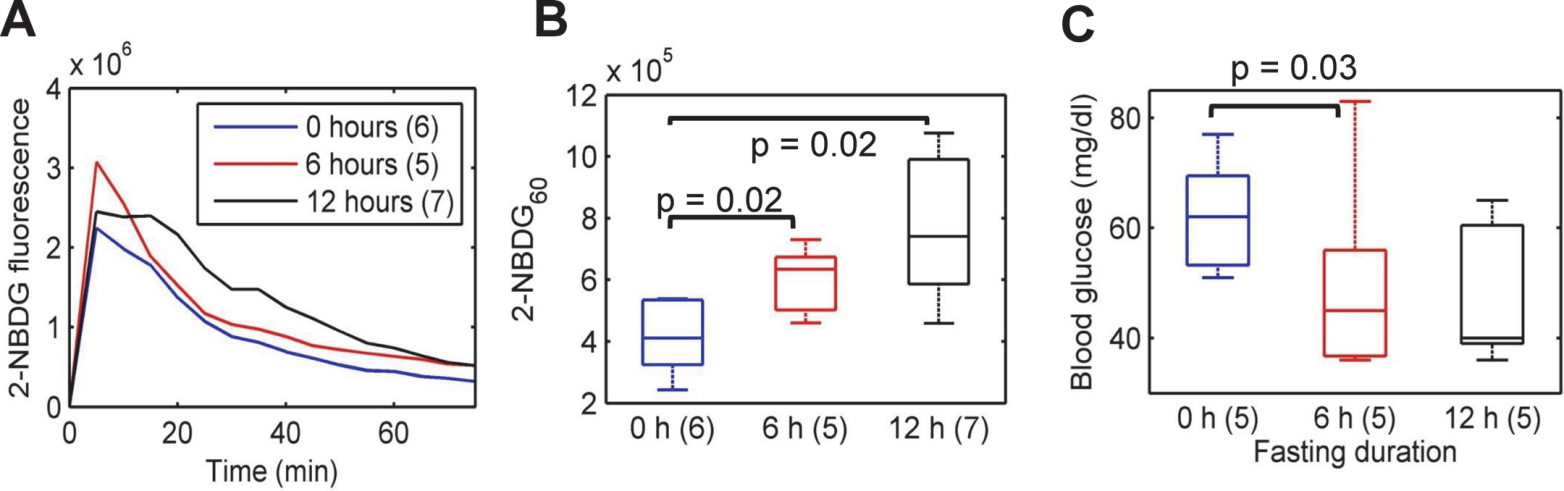
2-NBDG_60_ is sensitive to a decrease in blood glucose levels. **A**. Representative 2-NBDG kinetic profiles for 3 different fasting groups of mice after injection of a 6 mM (2mg/ml) dose via the tail-vein. There are no significant differences in maximum 2-NBDG-fluorescence between the different fasting groups, confirming that the delivery of 2-NBDG is similar across all animal groups. **B**. 2-NBDG_60_ is significantly higher in mice fasted for 6 hours and 12 hours compared with mice that were not fasted (p = 0.02). There are no significant differences between the 6 and 12-hour fasting groups. **C**. Blood glucose measurements were performed using a Freestyle Lite monitor by drawing 3 μl of blood from the tail of a separate cohort of mice (n = 5). Fasting for 6 hours led to a significant decrease in blood glucose levels. However, fasting for 12 hours did not lead to any further decrease in blood glucose levels. Statistical analysis was conducted using Wilcoxon rank sum tests.

**Fig. 4A** presents representative kinetic profiles of 2-NBDG uptake for three different periods of food deprivation in mice – 0, 6, and 12 hours. All measurements were made on non-tumor-bearing mice that were injected with a 6 mM (2 mg/ml) dose of 2-NBDG. Maximum 2-NBDG-fluorescence is similar in all three fasting groups (0, 6, and 12 hours). 2-NBDG_60_ is significantly higher in the 6-h and 12-h fasting groups compared with the 0-h fasting group (**Fig. 4B**; p = 0.02). However, there were no significant differences in fluorescence between the 6 and 12-h fasting groups. In a separate cohort of mice, we determined blood glucose levels for different fasting durations ranging from 0–12 hours. Mice fasted for 6 hours showed a statistically significant decrease in blood glucose levels relative to baseline blood glucose levels (**Fig. 4C**). Fasting for 12 hours did not lead to any further decrease in blood glucose levels. These data illustrate that changes in 2-NBDG_60_ for the different fasting durations are consistent with blood glucose dynamics over a similar time period.

### 2-NBDG_60_ increases with injected 2-NBDG dose

**Fig 5:**
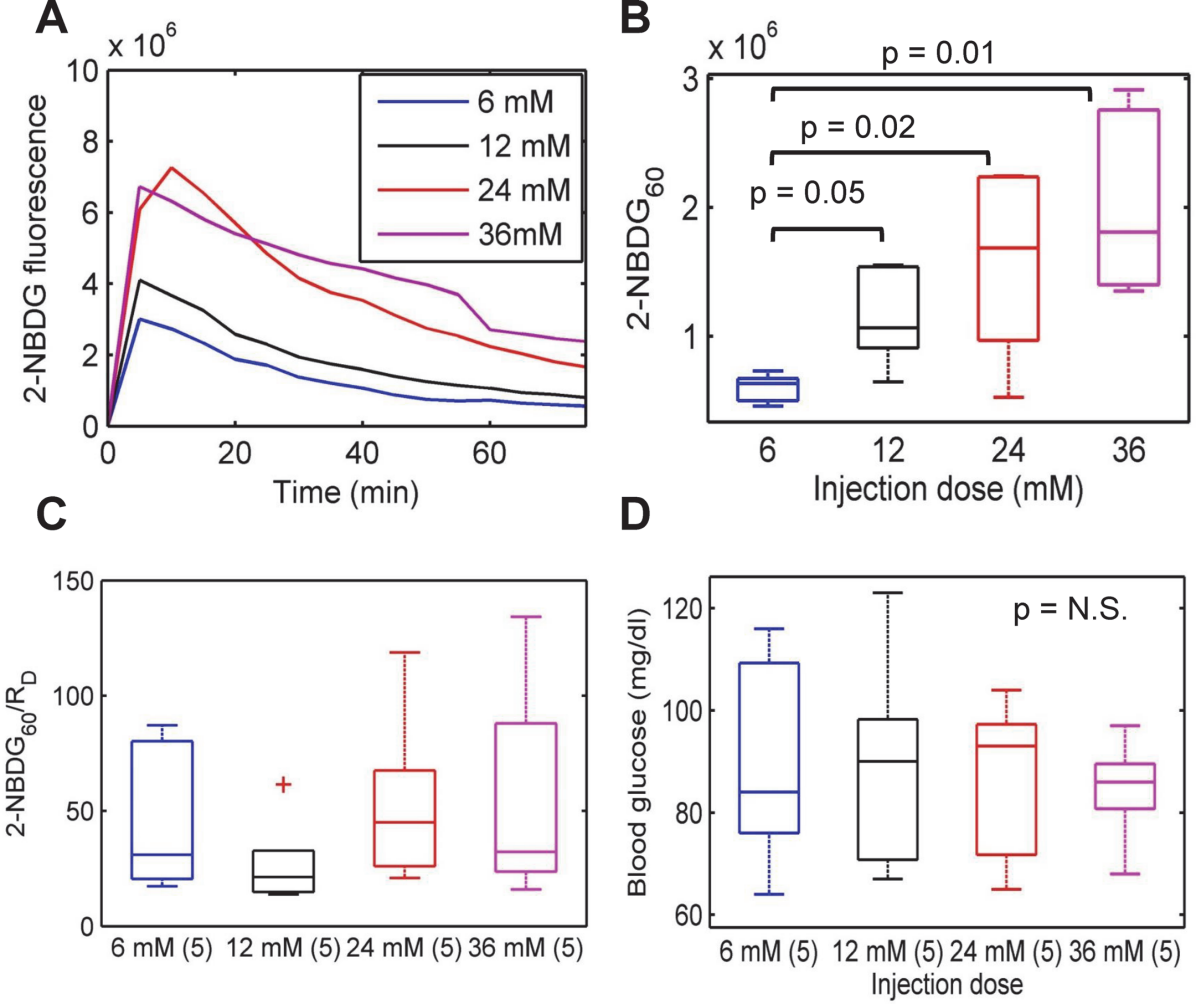
Optical spectroscopy is sensitive to changes in injected 2-NBDG dose. **A**. Representative kinetic profiles corresponding to the injection of increasing doses of 2-NBDG. Here, a 6 mM dose corresponds to 2 mg/ml of 2-NBDG or 8 mg/kg in mice. **B**. 2-NBDG_60_ for each dose greater than 6 mM is significantly higher compared with 2-NBDG_60_ for 6 mM. **C**. The ratio of 2-NBDG_60_/R_D_ corrects for changes due to injected dose and allows for comparison between pre-clinical studies that potentially use different doses. **D**. Blood glucose levels were not significantly different between the different dose groups. Statistical analysis was conducted using Wilcoxon sign rank tests for paired samples.

In addition to performing a ‘negative’ perturbation, we examined the effect of increasing the injected 2-NBDG concentration. **Fig. 5A** shows representative kinetic profiles of 2-NBDG uptake in normal, non-tumor bearing mice injected with increasing concentrations of 2-NBDG (6, 12, 24, and 36 mM). Injection of each dose greater than 6 mM caused a significant increase in maximum 2-NBDG-fluorescence as well as 2-NBDG_60_ (**Fig. 5B**) compared with the 6 mM dose. The rate of delivery (R_D_) of 2-NBDG is defined as the ratio of background-subtracted maximum 2-NBDG-fluorescence to the time taken to reach maximum fluorescence (2-NBDG_max_/T_max_). The ratio of 2-NBDG_60_/R_D_ essentially represents 2-NBDG uptake as a fraction of what was delivered in this particular context. There are no significant differences in 2-NBDG_60_/R_D_ between the different groups (**Fig. 5C)**. Analysis of the blood glucose levels that were measured prior to 2-NBDG injection found no significant differences between the different groups either suggesting the importance of correcting for dose dependent effects (**Fig. 5D**).

### 2-NBDG_60_ and SO_2_ measurements reveal an inverse relationship between glucose uptake and oxygen saturation in 4T1 murine mammary tumors

**Fig 6:**
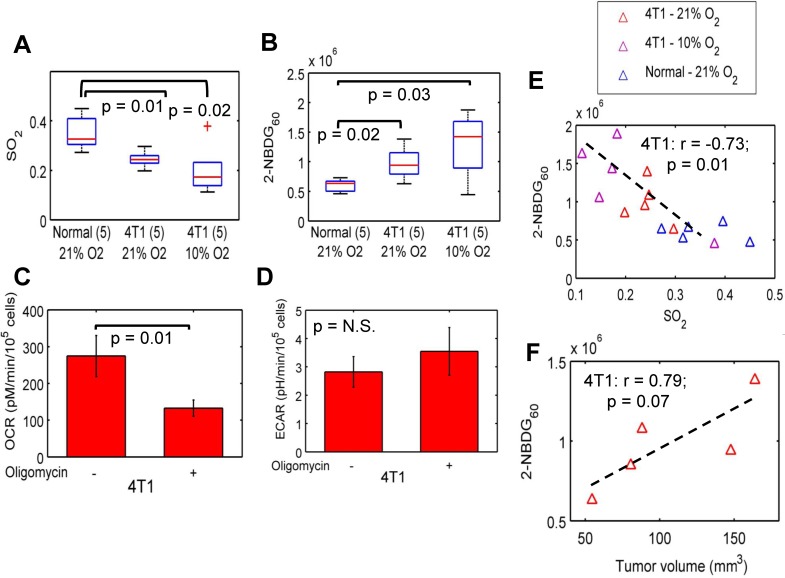
Optical spectroscopy reveals differences in glycolytic and vascular characteristics of 4T1 murine mammary tumors. **A**. SO_2_ is significantly lower in 4T1 (both normoxia and hypoxia) tumors compared with normal tissue. **B**. 2-NBDG_60_ is significantly higher in 4T1 tumors exposed to normoxia and hypoxia compared with normal tissue (p = 0.02 and 0.03, respectively). **C**. Blockade of respiration with oligomycin significantly reduces OCR in the 4T1 cells. **D**. However, there is only a small but insignificant increase in ECAR in response to oligomycin. Data represent n = 12 cell samples from 3 distinct assays. **E**. 2-NBDG_60_ is inversely correlated with SO_2_ for the 4T1 tumors that were exposed to 21% O_2_ and 10% O_2_ (r = -0.73; p = 0.01). Non-tumor-bearing mice, and 4T1 tumor-bearing mice that were breathing normoxia and hypoxia are shown here. 4T1 tumor-bearing mice were exposed to 10% oxygen (rest nitrogen) prior to 2-NBDG injection. **F**. 2-NBDG uptake in the 4T1 tumors is positively correlated with tumor volume (r = 0.79; p = 0.07). Error bars indicate standard error of the mean.

**Fig. 6A** and **6B** present the SO_2_ and 2-NBDG_60_ for the three tissue and tumor types—normal tissue, 4T1 tumor-bearing mice that were breathing room air (or 21% O_2_) and 4T1-tumor bearing mice that were breathing 10% O_2_ (rest nitrogen). SO_2_ is significantly higher in normal, non-tumor-bearing mice compared with all other groups. 2-NBDG_60_ is significantly higher in the 4T1 tumors exposed to 21% O_2_ and 10% O_2_ compared with normal tissue. The median SO_2_ of hypoxic 4T1 tumors was lower than their normoxic counterparts, but not statistically significant (Wilcoxon sign rank; p = 0.43). **Fig. 6C** and **6D** shows the results of a Seahorse Glycolytic Stress Test in 4T1 cells. After treatment with oligomycin, which prevents respiration, OCR decreased significantly in 4T1 cells (p<0.01). *In vivo*, we observe a similar decrease in SO_2_ after hypoxia. The decrease in OCR in 4T1 cells after treatment with oligomycin was accompanied by a small but insignificant increase in ECAR. **Fig. 6E** illustrates the relationship between the two parameters for all tissue types. When the 4T1 tumors were exposed to hypoxia, the median 2-NBDG_60_ increased for the hypoxic group; however, this increase was not statistically significant (Wilcoxon sign rank; p = 0.43). When the 4T1 tumors exposed to normoxia are considered along with the hypoxic group, 2-NBDG_60_ is inversely related with SO_2_ (r = -0.73; p = 0.01). An examination of 4T1 tumor volumes indicated a positive correlation between 2-NBDG_60_ and tumor volume (**Fig. 6F;** r = 0.79; p = 0.07).

## Discussion

The primary goal of our study was to demonstrate fast and non-invasive quantitative measurements of SO_2_ and glucose uptake via optical spectroscopy, to use these parameters to understand the differences in sibling cell lines that are derived from the same parental line but have different long-term outcome, and to compare them with normal non-tumor bearing tissue. The 4T1 and 4T07 are derived from a single spontaneous mammary tumor grown in Balb/cfC_3_H mice [36]; although both cell lines are highly tumorigenic, the 4T1 is highly metastatic and the 4T07 is capable of only systemic invasion [36]. Knowledge of the oxygenation and metabolic properties of 4T1 and 4T07 cells is derived mostly from *in vitro* metabolic [41] and metabolomic assays [42]. Our interest in metastatic outcome stems from recent studies on tumor metabolism that indicate an inclination towards aerobic glycolysis in aggressive cell lines and tumors [11–13,43].

One of the most important requirements of an externally injected compound that reports on tissue function is that the reporter should not affect the existing tissue environment. Snyder et al. have previously observed that injection of glucose at concentrations ranging from 1–4 g/kg of body weight in rats significantly changed tissue pO_2_ [44]. The dose of 2-NBDG used in this study was 8 mg/kg. We found no significant change in SO_2_ after 2-NBDG-administration and no correlation between the fold-increase of SO_2_ and 2-NBDG_60_ in normal tissue and tumors. Furthermore, doses of up to 64 mg/kg were evaluated for their effect on SO_2_ and no significant dose-specific effects were found. The baseline, pre-injection SO_2_ measurements that were extracted from the reflectance spectra were significantly lower in the 4T1 and 4T07 tumors. These results are consistent with previous studies that have established that solid tumors have lower levels of oxygenation (or pO_2_) due to increased cellular demand and the development of hypoxic regions due to disordered vasculature [45,46].

2-NBDG-fluorescence spectra measured in tumors, specifically 4T1 xenografts, showed significant distortion in the wavelength region corresponding to hemoglobin absorption. Correction with the MC fluorescence model removed the spectral distortion and recovered a 2-NBDG-fluorescence line shape that was in good agreement with the true line shape of 2-NBDG that was measured in solution. Due to significantly lower hemoglobin absorption, the 2-NBDG-fluorescence measured from normal tissue did not contain significant spectral distortions. After correction with the MC fluorescence model, 2-NBDG_60_ was significantly higher in the 4T1 murine mammary tumor xenografts compared with normal tissue. This result is consistent with the fact that rapidly proliferating tumors are highly glucose-avid. The 2-NBDG uptake in 4T07 tumors was highly variable and not distinctly different from that of the 4T1 tumors or the normal, non-tumor bearing tissues. For the 4T07 study alone, we also had data from the contralateral ‘non-tumor’ flank. We found that 2-NBDG-uptake in the 4T07 tumors with the lowest 2-NBDG uptake was lower than the contralateral normal side. Glucose uptake in tumors is dependent on the availability of substrate and can be affected by changes in blood flow and delivery [23,47–49]. Thus, we speculate that this result can be attributed to either poor delivery of the tracer or a delivery rate in excess of the glucose consumption rate. However, further studies will be required to fully understand the basis for the high variability observed in the 4T07 tumors.

In a previous study using the same cell lines in dorsal skin-fold window chambers, we observed that for the same average tumor size, SO_2_ of the 4T07 tumors was significantly higher compared with SO_2_ of the 4T1 tumors [23]. It must be noted that the vascular length density for both 4T1 and 4T07 tumors were statistically similar in the window chambers, and this enabled a direct comparison of their SO_2_ profiles *in vivo*. In the current study, tumor volumes (Wilcoxon rank sum: p = 0.69) and SO_2_ of both cell lines were statistically similar to each other. Furthermore, [THb] which can be used as a surrogate measure of tissue vascularity was statistically similar between the 4T1 and 4T07 tumors. The 4T07 tumors grown in window chambers showed little to no 2-NBDG uptake at high oxygenation levels whereas the 4T1 tumors exhibited significantly higher 2-NBDG uptake at similarly high and significantly lower oxygenation conditions (aerobic glycolysis). However, there were no regions with low SO_2_ (<0.2) in the 4T07 tumors to evaluate its response to low-oxygen conditions. In the current study, we observed that 2-NBDG uptake was indeed high in the 4T07 tumors at low SO_2_ levels, and that there were no significant differences in 2-NBDG-uptake in the 4T1 and 4T07 tumors under low SO_2_ levels (0.2<SO_2_<0.3). Metabolic analysis of the 4T1 and 4T07 cells revealed no significant differences in either OCR or ECAR for both cell lines either.

‘Food-deprivation’ or fasting overnight for 8–10 hours prior to imaging with PET is required to significantly enhance uptake by and hence contrast from potential tumors located in the human body. Fasting for 6 hours during the day led to a significant increase in 2-NBDG_60_ compared with the 0-h fasting group. 2-NBDG_60_ in the 12-hour fasting group was not significantly higher than the 6-hour fasting group. It is important to note that the 12-hour fasting was conducted overnight. Mice have a higher metabolic rate compared with humans and are nocturnal feeders, with approximately 70% of their daily consumption occurring at night [50]. Therefore, fasting overnight could have significantly affected their blood glucose levels, causing them to draw on their glycogen reserves to provide energy. These results could potentially explain why 2-NBDG_60_ in mice fasted for 12 hours overnight is not significantly higher than shorter fasting durations. Although measurements of blood glucose in mice for different periods of fasting indicate that fasting for 10 hours causes the lowest levels of blood glucose relative to baseline, these levels are not significantly lower than that for a 6-hour fast. A 6-hour fast during the day is also feasible in terms of logistics and performing studies on multiple animals on a single day. These data demonstrate that an increase in 2-NBDG_60_ is inversely related to blood glucose levels.

Next, we determined the sensitivity of *in vivo* 2-NBDG uptake to increasing concentrations of 2-NBDG. The purpose of this experiment was to examine glucose uptake in response to a perturbation that did not affect blood glucose levels but delivered varying amounts of 2-NBDG molecules to tissue. We found a linear increase in 2-NBDG_60_ with increasing concentrations of 2-NBDG. Increasing the injected concentration of 2-NBDG also increases R_D_, which is calculated based on the maximum fluorescence observed in tissue. In this context of varying dose, R_D_ becomes a surrogate endpoint that represents the dose and allows for calibration of the 2-NBDG_60_. Specifically, data that are obtained using different doses of 2-NBDG in the same tissue type can be compared by calibrating the 2-NBDG_60_ by R_D_. There were no significant differences in 2-NBDG_60_/R_D_ for different doses, and this was consistent with the similar blood glucose levels for all doses.

When we exposed the 4T1 tumors to hypoxia to further decrease tumor oxygenation, 2-NBDG_60_ of the 4T1 tumors increased relative to both baseline and to the 4T07 tumors. We observed a significant negative correlation between 2-NBDG_60_ and SO_2_ for the 4T1 (normoxia and hypoxia) tumors (r = -0.73; p = 0.01). This result was consistent with the Pasteur effect or anaerobic glycolysis. The observation of a correlation indicating anaerobic glycolysis in this study is interesting when considered in the context of the previous study in dorsal window chambers. We previously observed that 2-NBDG_60_ across the entire tumor (whole-tumor intravital imaging) was elevated at all SO_2_ levels (>0.2) in the 4T1 tumors, and there was no SO_2_-dependent change in 2-NBDG_60_. Blockade of mitochondrial respiration in 4T1 cells significantly decreased OCR but did not cause a significant increase in ECAR either. The ECAR results are consistent with the dorsal skin flap window chamber model; however, neither of these results is consistent with the results reported from the solid 4T1 tumors in the current study. One likely explanation for this discrepancy is the large variance in the solid 4T1 tumors in this study because they are not constrained by a window. In fact, 2-NBDG_60_ in the solid 4T1 tumors is positively correlated with tumor size. In normal tissue, we did not observe a correlation between SO_2_ and 2-NBDG_60_, and this could be attributed to the high SO_2_ levels seen in normal tissue. Kasishcke et al. showed recently that NADH, a metabolic coenzyme integral to glycolysis, was constant for pO_2_ levels in the mouse cortex > 10 mmHg but increased steeply for pO_2_ < 10 mmHg, indicating increased glycolysis [51].

In summary, we have presented a non-invasive and fast optical strategy for simultaneously quantifying SO_2_ and glucose uptake *in vivo*. Our results illustrate the potential for a valuable pre-clinical and clinical tool to noninvasively quantify the relationship between SO_2_ and glucose uptake. Our eventual goal is to determine the ability of combined measurements of SO_2_ and glucose uptake in the primary tumor to provide biomarkers of resistance and metastatic potential.
